# MiRNA-132 regulates the development of osteoarthritis in correlation with the modulation of PTEN/PI3K/AKT signaling

**DOI:** 10.1186/s12877-021-02046-8

**Published:** 2021-03-10

**Authors:** Wei Zhang, Chengfang Hu, Chi Zhang, Congfeng Luo, Biao Zhong, Xiaowei Yu

**Affiliations:** Department of Orthopaedics, Shanghai Jiao Tong University Affiliated Sixth People’s Hospital, No. 600 Yishan Road, Shanghai, 200233 China

**Keywords:** miR-132, Osteoarthritis, Chondrocytes, PTEN/PI3K/AKT signaling pathway

## Abstract

**Background:**

Osteoarthritis (OA) is a commonly known prevalent joint disease, with limited therapeutic methods. This study aimed to investigate the functions of miRNA-132 (miR-132) in the modulation of PTEN/PI3K/AKT signaling pathway in the development and progression of osteoarthritis.

**Methods:**

Eight male osteoarthritic patients and eight healthy males were recruited. Male Sprague Dawley (SD) rats were used for cellular experiments. QRT-PCR was performed to detect the expression levels of miR-132, PTEN, PI3K and AKT. MTT assay and apoptosis assay were carried out to measure the cell proliferation rate and cell apoptosis rate, respectively. Western blotting was employed to detect the protein expression of related RNAs and inflammatory factors.

**Results:**

In osteoarthritic patients, the expression level of miR-132 was decreased, compared with that in the normal group. Over-expression of miR-132 elevated cell proliferation and decreased apoptosis of chondrocytes. Down-regulation of miR-132 decreased cell proliferation and induced apoptosis in chondrocytes. In addition, down-regulation of miR-132 promoted the expression of Bax protein and activated caspase-3/9, increased inflammation divisors. PTEN inhibitor antagonized the destructive effect of the miR-132 inhibitor on cell proliferation of chondrocytes. PI3K inhibitor increased the destructive effect of the miR-132 inhibitor on osteoarthritis.

**Conclusion:**

In conclusion, miR-132 is an important regulator of osteoarthritis in chondrocytes through the PTEN/PI3K/AKT signaling pathway.

## Background

Osteoarthritis (OA) is a commonly known prevalent joint disease accompanied by heavy pain, function loss, and even disabilities in adults, especially in elderly people [1–3]. OA is usually resulted from obesity, major injury, as well as heavy labor and frequent knee bending [4–6]. Although radiographic detection and physical examinations are available, the diagnostics and prognostics of OA are still unsatisfied [7]. Due to the complexity and difficulty with early diagnostics, there are no effective treatments to prevent or manage the development of OA [8, 9]. The pathophysiology and pathogenesis of OA remain unclear. Therefore, discoveries of more reliable biomarkers and therapeutic agents are urgently needed.

MiRNAs form a big group of small non-coding RNAs with 19–23 nucleotides, which could bind to the 3′-UTR region of corresponding messenger RNAs (mRNAs) to suppress their protein expression [10–12]. MiRNAs have been observed to implicate in various cellular processes, such as cell apoptosis, lipid metabolisms, malignant transformations and cell differentiation [13–15]. For osteoarthritis, accumulative evidence suggests that many types of miRNAs could alleviate progressions of osteoarthritis, such as miR-140 [16], miR-130a [17], miR-16-5p [18] and so on [19–21]. One study proposed that miR-130a acted as a regulator in the expression of TNF-α in human chondrocytes and miR-130a was identified as a potential inhibitor for OA [17]. MiR-132 has been demonstrated to have regulatory effects in antiviral innate immunity [22] and pancreatic cancer [23]. It was observed that the expression levels of miR-132 were decreased in osteoarthritic patients compared with that in healthy controls [24]. Another study also reported the potential of miR-132 in regulating the progression of osteoarthritis [24].

The PTEN/PI3K/AKT signaling pathway was reported to regulate the signaling of various biological processes such as cell apoptosis, proliferation and growth [25–27]. PTEN (phosphatase and tensin homolog deleted on chromosome ten) is a dual protein/lipid phosphatase, which regulated the downstream molecules of PI3K/AKT pathway [28]. The activation of the PI3K/AKT pathway could help prevent cell apoptosis and promote cell proliferation [29]. PTEN/PI3K/AKT pathway was demonstrated to impact the pathological development of glioma tumor [26] and the viability of prostate cancer stem-like cells [30]. More importantly, it was well established that the PTEN/PI3K/AKT pathway was targeted by miR-214 and affected osteoclast-genesis [28]. In this study, we aimed to investigate the role of miR-132, and the interactions between miR-132 and the PTEN/PI3K/AKT signaling pathway in the progression of osteoarthritis. Our findings may provide novel insights into the prevention or therapeutic approaches of osteoarthritis.

## Methods

### Patients

Eight male osteoarthritic patients with an average age of 52.46 ± 5.18 years old and eight healthy males with an average age of 54.72 ± 6.05 years old were recruited from the Shanghai Jiao Tong University Affiliated Sixth People’s Hospital during total knee replacement surgeries. Peripheral blood (10 mL) was taken and centrifuged at 2000 g at 4 °C for 10 min. The serum was stored at − 80 °C.

### Cell isolation and treatment

Male Sprague Dawley (SD) rats weighted from 220 to 250 g and aged 8–9 weeks old were purchased from Shanghai Slick Experimental Animal Co., Ltd., China. Rats were maintained at 22.5 °C with 58% humidity. The rats were anesthetized by sodium pentobarbital (30 mg/kg body weight, Sigma Chemical Co., St. Louis, MO, USA) intraperitoneally and euthanized by cervical dislocation. The cartilage tissues were taken, washed, sterilized and sliced. Tissues were digested by 0.25% Trypsin-EDTA for 30 min and collagenase II (Invitrogen, USA) for 4 h on the ice, and filtered by the 200-mesh sieve. Chondrocytes were cultured with DMEM with 4.5 g/l glucose, 10% FBS, and 1% penicillin/streptomycin at 37 °C with 5% CO_2_. The miR-132 mimics (5′-UAACAGUCUACAGCCAUGGUCG-3′), negative controls (NC) (5′-UCACAACCUCCUAGAAAGAGUAGA-3′), miR-132 inhibitor (5′-CGACCATGGCTGTAGACTGTTA-3′) and inhibitor NC (5′-GTGTAACACGTCTATACGCCCA-3′) were obtained from Sangon Biotech Co., Ltd., Shanghai, China. Lipofectamine 2000 reagent (Invitrogen, USA) was used for transfection. VO-Ohpic trihydrate (10 nM, PTEN inhibitor) or wortmannin (2 nM, PI3K inhibitor) were added to cells for 72 h.

### qRT-PCR

Total RNAs were extracted from serum and cells by TRIzol reagent. RNA samples were treated by DNase I (Invitrogen, USA) to remove genomic DNAs. Superscript II reverse transcriptase (Invitrogen, USA) and oligo (dT)_20_ were used to reverse transcribe RNAs into cDNAs. RT-PCR was performed in a StepOnePlus Real-time PCR system (Applied Biosystems, USA). PCR was performed at 95 °C for ten min, 40 cycles of 94 °C for 30 s, 55 °C for 30 s, 60 °C for 10 s, and 72 °C for 30 s. was used. The expression levels of miR-132 were determined with U6 as the internal reference. The primers used were listed as following: 5′-TGGATCCCCCCCAGTCCCCGTCCCTCAG-3′ (forward) and 5′-TGAATTCGGATACCTTGGCCGGGAGGAC-3′ (reverse) for miR-132; 5′-CTCGCTTCGGCAGCACA-3′ (forward) and 5′-AACGCTTCAGAATTTGCGT-3′ (reverse) for U6.

### Cell viability assays

After transfection, 10 μl 3-(4, 5-Dimethylthiazol-2-yl)-2, 5-diphenyltetrazolium bromide (MTT) (5 mg/ml; Invitrogen) was pipetted and incubated at 37 °C for 4 h. After removing the culture medium, dimethyl sulphoxide (150 μl; Invitrogen, USA) was added to each well and incubated at room temperature for 3 h in the dark. The absorbance at 570 nm was measured.

### Apoptosis assay and ELISA test

After inhibitor treatment, cells were washed, resuspend and stained by 5 μl Annexin V-FITC and 5 μl propidium iodide (PI) (BD Biosciences) at 25 °C for 15 min in the dark. Flow cytometry (FACSCanto™) was used to measure apoptosis rate, which was analyzed by CellQuest Pro (BD Biosciences). Supernatants were collected to detect IL-1β, IL-6 and IL-18 via ELISA kit from Jiancheng Biology, China. Multiskan Go Microplate Spectrophotometer (Thermo Fisher Scientific) measured the absorbance at 450 nm.

### Western blotting

After transfection, cells were lysed and centrifuged at 12,000 rpm at 4 °C for 10 min. Proteins were resolved on SDS-PAGE, transferred to PVDF membranes (Millipore, USA), and blocked by 5% non-fat milk. Membranes were then incubated with primary antibodies against: Bax (1:500, 2774; CST, USA), cleaved Caspase-3 (1:500, 9661; CST, USA), cleaved Caspase-9 (1:500, 20,750; CST, USA), PTEN (1:500, 9552; CST, USA), PI3K (1:500, 4255; CST, USA), p-AKT (1:500, 4060; CST, USA) and GAPDH (1:2000, 5174; CST, USA) at 4 °C for overnight. After wash, HRP-linked anti-rabbit secondary antibodies (1:5000, 7074; CST, USA) were incubated with the membrane. Protein signals were detected by chemiluminescence kit and analyzed by AlphaEaseFC 4.0.

### Statistical analysis

Statistical analysis was conducted by SPSS 17.0. Data were presented as mean ± SD. T-test was utilized for comparisons between two groups. All experiments were repeated for 3 times. The one-way ANOVA and Bonferroni’s post-hoc tests were carried out to explore the differences among multiple groups. *P* < 0.05 was considered to be significant difference.

## Results

### Effects of miR-132 on cell proliferation and apoptosis of chondrocytes

**Fig. 1:**
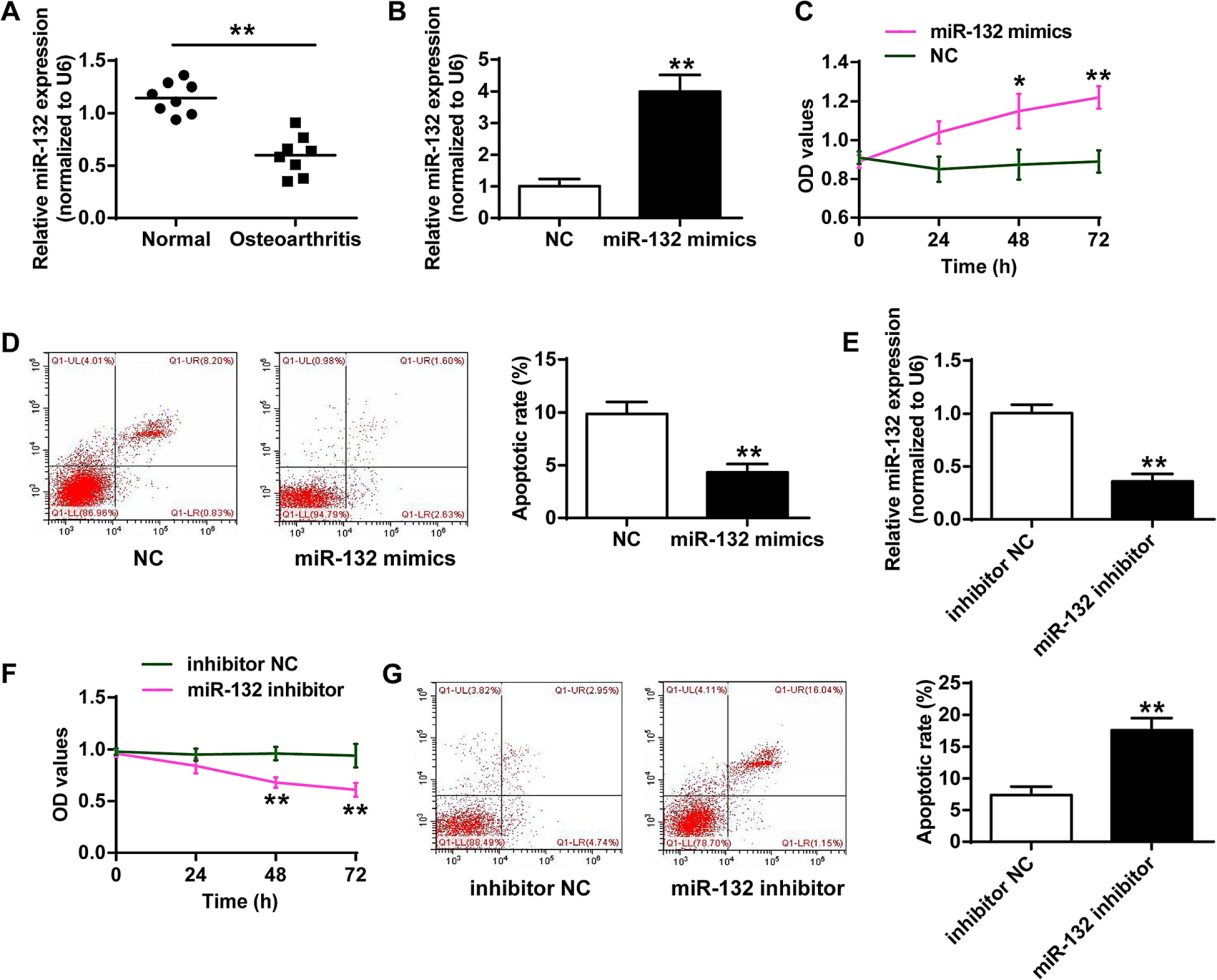
Effects of miR-132 on cell proliferation and apoptosis of chondrocytes. **a** The expression of miR-132 in osteoarthritic patients. **b** qRT-PCR for relative expression of miR-132 in chondrocytes after transfection of miR-132 mimics. **c** Cell proliferation of chondrocytes after transfection of miR-132 mimics. **d** Chondrocytes cell apoptosis rate of after transfection of miR-132 mimics. **e** qRT-PCR for the expression of miR-132 in chondrocytes after transfection of miR-132 inhibitor. **f** Cell proliferation of chondrocytes after transfection of miR-132 inhibitor. **g** Cell apoptosis of chondrocytes after transfection of miR-132 inhibitor. NC: negative control. *N* = 4 per group. *P < 0.05, **P < 0.01

To identify the function of miRNA-132 in the development and progression of osteoarthritis, the expression of miRNA-132 was evaluated by qRT-PCR. As shown in Fig. 1A, the expression levels of miR-132 were down-regulated in the serum of OA patients, compared with that in the normal group (*P* < 0.01). In addition, miR-132 mimics effectively elevated the expression of miR-132 (Fig. 1B), suggesting the successful transfection of miR-132. Over-expression of miR-132 increased cell proliferation (Fig. 1C) and inhibited cell apoptosis of chondrocytes (Fig. 1D) (*P* < 0.05, *P* < 0.01). As shown in Fig. 1E, miR-132-inhibitor suppressed the expression of miR-132, suggesting the successful transfection of miR-132 inhibitor (*P* < 0.01). Furthermore, transfection of miR-132 inhibitor also repressed cell proliferation and induced chondrocytes cell apoptosis (*P* < 0.01) (Fig. 1F and G).

### Effects of miR-132 inhibitor on the expression of Bax and caspase-3/9 protein and inflammation divisors in chondrocytes

**Fig. 2:**
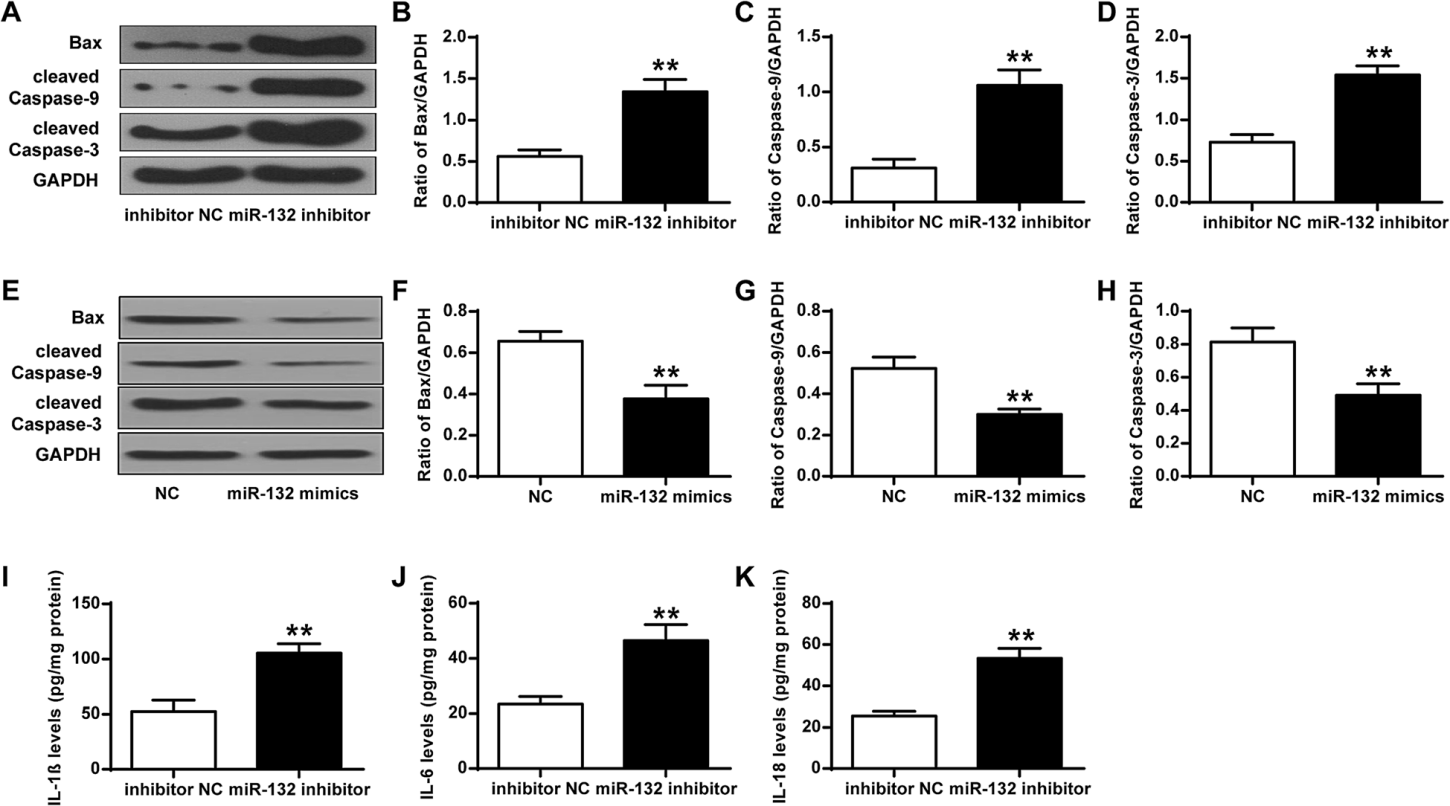
Effects of miR-132 inhibitor on Bax and caspase-3/9 protein expression and inflammation divisors in chondrocytes. **a** Representative western blot bands of Bax, cleaved Caspase-9, cleaved Caspase-3, and GAPDH. **b** Quantitative densitometry of the expression of Bax protein after transfection of inhibitor NC and miR-132-inhibitor. **c** Quantitative densitometry of the expression of Caspase-9 protein. **d** Quantitative densitometry of the expression of Caspase-3 protein. **e** IL-1β levels in cells transfected with inhibitor NC and miR-132-inhibitor. **f** IL-6 levels in cells transfected with inhibitor NC and miR-132-inhibitor. **g** IL-18 levels in cells transfected with inhibitor NC and miR-132-inhibitor. Pg/mg means contents of IL-1β/IL-6/ IL-18 per mg of proteins. **P < 0.01

Western blotting was performed to examine the effects of miR-132 inhibitor on the the expression of inflammation related proteins. It showed that down-regulation of miR-132 markedly promoted the expression of Bax and caspase-3/9 in chondrocytes (*P* < 0.01) (Fig. 2A-D). Also, the expression of BAX, Bcl-2, cleaved caspase-3 and cleaved caspase-9 after adding miR-132 mimics to chondrocytes presented similar trend (Fig. 2E-H). In addition, the expression levels of IL-1β, IL-6 and IL-18 in chondrocytes were significantly increased by down-regulation of miR-132, but not in the negative control group (*P* < 0.01) (Fig. 2I-K). These results indicated that downregulation of miR-132 could enhance the expression of inflammation related protein.

### Down-regulation of miR-132 upregulated the expression of PTEN in chondrocytes

**Fig. 3:**
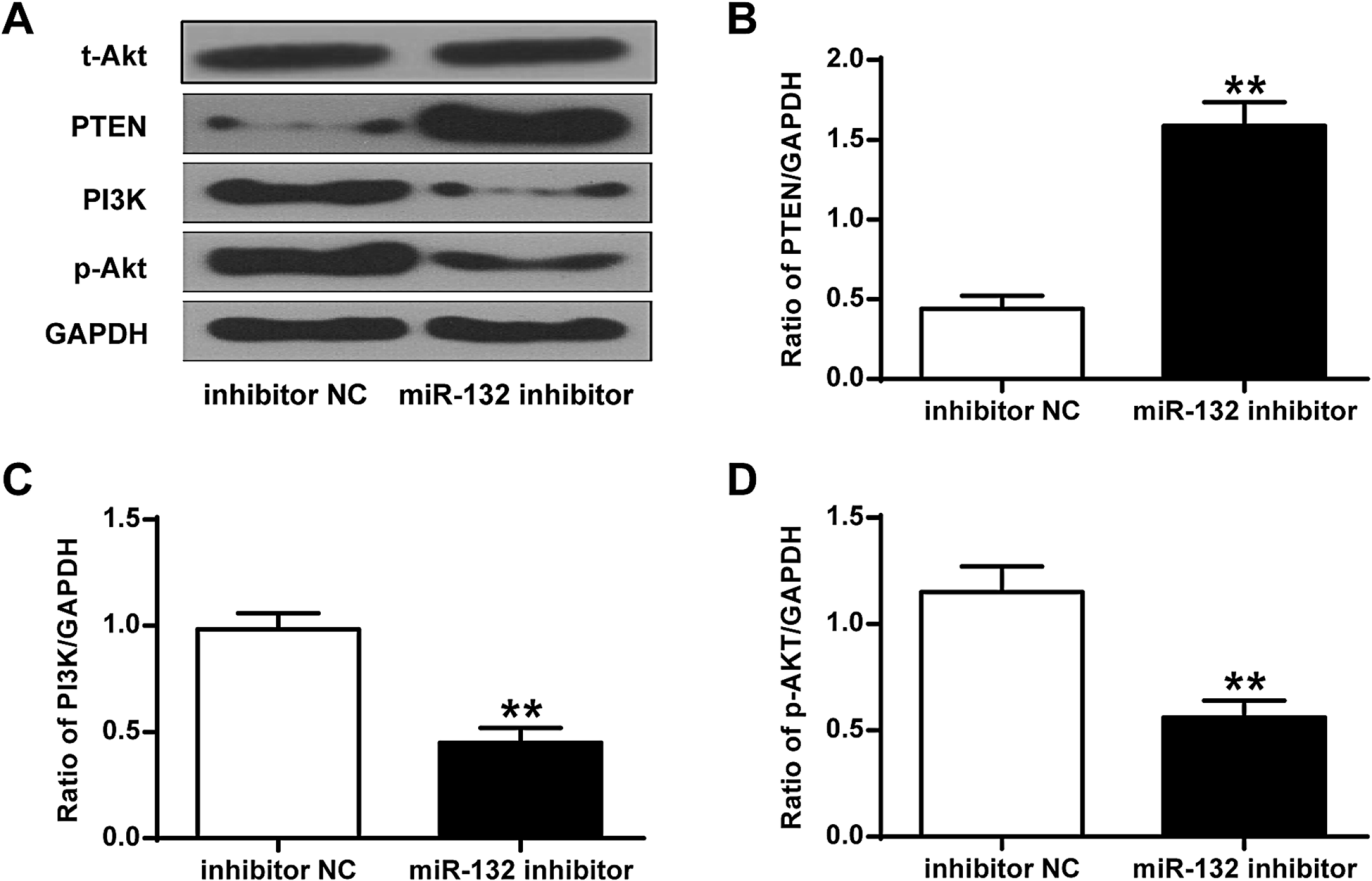
Down-regulation of miR-132 activated PTEN/PI3K/AKT signaling pathway in chondrocytes. **a** Representative western blot bands for protein expression of PTEN, PI3K, p-AKT, and GAPDH. **b** The ratio in the expression of PTEN versus GAPDH. **c** Ratio in the expression of PI3K versus GAPDH. **d** Ratio in the expression of p-AKT versus GAPDH. **P < 0.01

To further investigate the role of the PTEN/PI3K/Akt signaling pathway on the regulation of microRNA-132 in osteoarthritis, miR-132 was knocked down. As shown in Fig. 3A-D, down-regulation of miR-132 greatly elevated the expression of PTEN, and inhibited the expression of PI3K and p-AKT in chondrocytes when compared with that in the negative control (*P* < 0.01).

### PTEN inhibitor antagonized the destructive effect of miR-132 inhibitor on PTEN/PI3K/AKT signaling pathway, cell proliferation, and inflammation divisors of chondrocytes

**Fig. 4:**
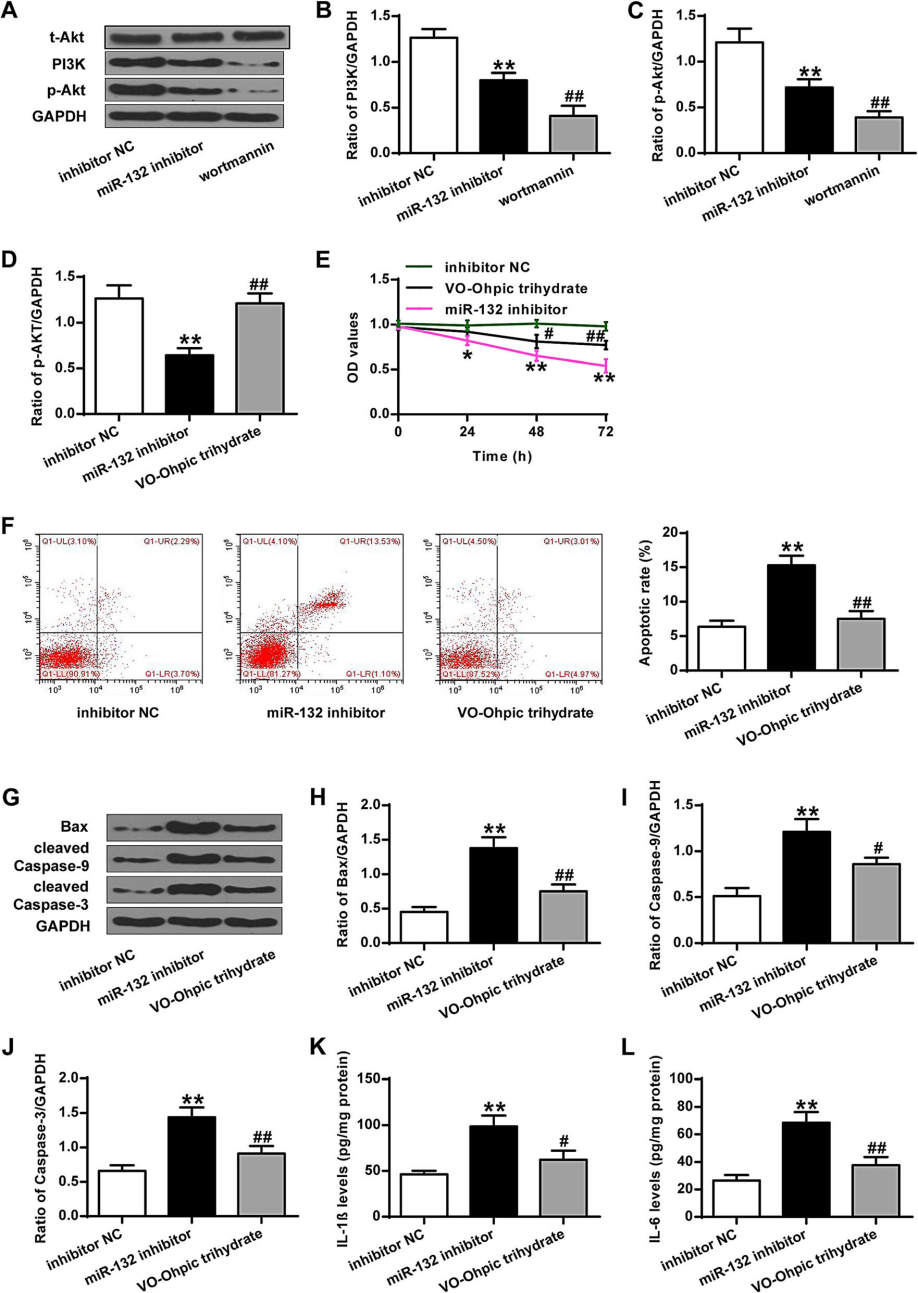
PTEN inhibitor antagonized the destructive effect of the miR-132 inhibitor on PTEN/PI3K/AKT signaling pathway, cell proliferation, and inflammation divisors of chondrocytes. **a** Representative western blot bands of PTEN, PI3K, p-Akt, and GAPDH in control, miR-132 inhibitor and miR-132 inhibitor +VO-Ohpic trihydrate group. **b** Quantitative densitometry of the expression of PTEN protein. **c** Quantitative densitometry of the expression of PI3K protein. **d** Quantitative densitometry of the expression of the p-AKT protein. **e** Cell proliferation of chondrocytes. **f** Apoptosis of chondrocytes. **g** Representative western blot bands of Bax, cleaved Caspase-9, cleaved Caspase-3, and GAPDH. **h** Quantitative densitometry of the expression of Bax protein. **i** Quantitative densitometry of the expression of Caspase-9 protein. **j** Quantitative densitometry of the expression of Caspase-3 protein. **k** IL-1β levels. **l** IL-6 levels. **m** IL-18 levels. VO-Ohpic trihydrate is the PTEN inhibitor. **P < 0.01 vs inhibitor NC group; #*P* < 0.05, ##*P* < 0.01 vs miR-132 inhibitor group

VO-Ohpic trihydrate, PTEN inhibitor, was used to inhibit the expression of PTEN in chondrocytes after downregulation of microRNA-132. It showed that PTEN inhibitor significantly inhibited the expression of PTEN, and resulted in upregulated expression of PI3K and p-AKT after down-regulation of miR-132 (*P* < 0.01) (Fig. 4A-D). It also showed that PTEN inhibitor greatly enhanced cell proliferation, inhibited cell apoptosis, and suppressed the expression of Bax and caspase-3/9 in chondrocytes after down-regulation of miR-132 (*P* < 0.05, *P* < 0.01) (Fig. 4E-J). As shown in Fig. 4K-M, PTEN inhibitor markedly decreased the expression of IL-1β, IL-6 and IL-18 in chondrocytes after down-regulation of miR-132 (*P* < 0.05, *P* < 0.01).

### Wortmannin increased the destructive effect of miR-132 inhibitor on PI3K/AKT signaling pathway, cell proliferation, and inflammation divisors of chondrocytes

**Fig. 5:**
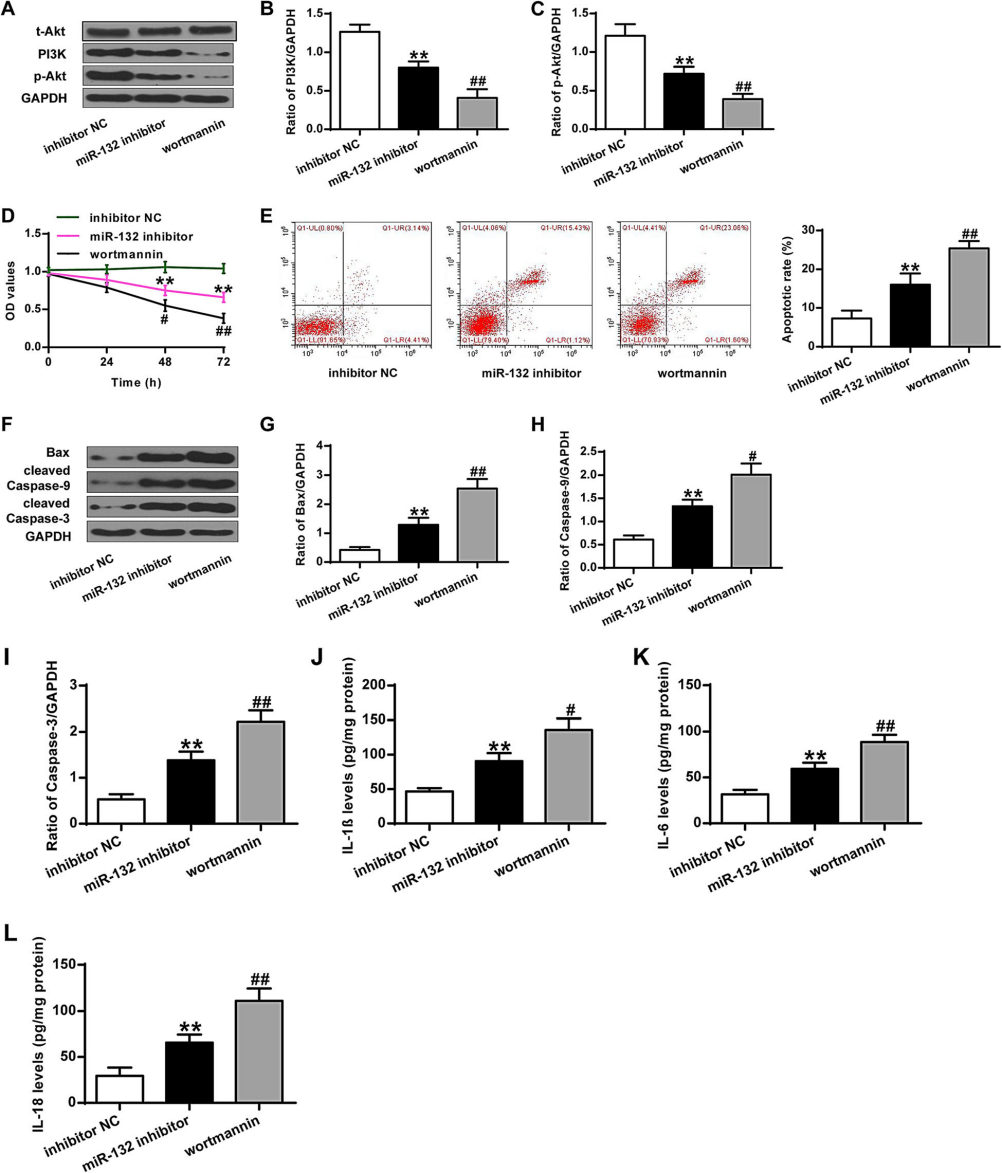
Wortmannin increases the destructive effect of the miR-132 inhibitor on PI3K/AKT signaling pathway, cell proliferation, and inflammation divisors of chondrocytes. **a** Representative western blot bands of PI3K, p-Akt, and GAPDH. **b** Quantitative densitometry of the expression of PI3K protein. **c** Quantitative densitometry of the expression of the p-AKT protein. **d** cell proliferation of chondrocytes. **e** Apoptosis of chondrocytes. **f** Representative western blot bands of Bax, cleaved Caspase-9, cleaved Caspase-3, and GAPDH. **g** Quantitative densitometry of the expression of Bax protein. **h** Quantitative densitometry of the expression of Caspase-9 protein. **i** Quantitative densitometry of the expression of Caspase-3 protein. **j** IL-1β levels. **k** IL-6 levels. **l** IL-18 levels. Wortmannin is the PI3K inhibitor. **P < 0.01 vs inhibitor NC group; #*P* < 0.05, ##*P* < 0.01 vs miR-132 inhibitor group

To further explore the molecular mechanisms underlying the suppressive effect of miR-132 on osteoarthritis, PI3K inhibitor was used to assess its function and mechanisms. As shown in Fig. 5A-C, wortmannin, PI3K inhibitor, greatly inhibited the expression of PI3K and p-AKT in chondrocytes after the down-regulation of miR-132 (*P* < 0.01). In addition, the suppression of PI3K significantly reduced cell proliferation, elevated cell apoptosis, and induced Bax and caspase-3/9 protein expression in chondrocytes after the down-regulation of miR-132 (*P* < 0.05, *P* < 0.01) (Fig. 5D-I). The suppression of PI3K obviously increased inflammation divisors in chondrocytes after down-regulation of miR-132 (*P* < 0.05, *P* < 0.01) (Fig. 5J-L). Overall, the PI3K inhibitor enhanced the functions of miR-132 on inflammation in osteoarthritis.

## Discussion

Osteoarthritis is a painful disease of articulating joints, with increasing prevalence all over the world. The initiation and progression of OA involve complex multi-factorial results from genetic mutations, mechanical stresses, and environmental factors. As the early diagnostics and treatment for OA are not ideal, searching for more sensitive biomarkers and reliable therapeutic targets are quite necessary. In this study, we evaluated the role of miR-132 and its interactions with PTEN/PI3K/AKT signaling pathway in the regulation of OA.

Studies have revealed that miRNAs are involved in the regulation/mediation of in cell viability, proliferation [31]. For instance, the silencing of miR-34a was observed to inhibit chondrocyte cell apoptosis in a rat osteoarthritis model in vitro [32]. In our study, we found that the expression levels of miR-132 were down-regulated, compared with the normal group. Over-expression of miR-132 elevated cell proliferation and inhibited cell apoptosis of chondrocytes, while down-regulation of miR-132 also repressed cell proliferation and induced chondrocytes cell apoptosis. Our studies confirmed that miR-132 correlated positively with cell proliferation, and negative with cell apoptosis.

It was observed that some miRNAs could regulate the expression of inflammation factors in osteoarthritides, such as miR-146a [33], miR-142-3p [34] and miR-130a [17]. One study reported that miR-146a regulated pain-associated inflammatory factors in human knee joint synoviocytes [33]. Our Western blotting results revealed that the down-regulation of miR-132 greatly enhanced the expression of Bax and caspase-3/9, as well as the inflammation factors in chondrocytes, comparing to the negative control group. It was well established that down-regulation of miR-132 can lower the protein expression of Bax and caspase-3/9 and inflammation divisors in chondrocytes.

Previous studies have shown that the PTEN/PI3K/AKT signaling pathways actively participate in the regulation of many kinds of human cancer [25] and osteoclast-genesis [28]. In addition, the dysregulations of specific miRNAs could impact the activation or inactivation of this pathway, including miR-214 [28], miR-21 [35] and miR-519a [36]. To the best of our knowledge, we are the first to investigate the interactions between miR-132 and the PTEN/PI3K/AKT pathway in OA. Our Western blotting results and the ratio analysis strongly suggest that down-regulation of miR-132 greatly elevates the expression of PTEN and inhibits the expression of PI3K and p-AKT in chondrocytes. Also, down-regulation of miR-132 elevated the expression of PTEN, while PETN inhibitor resulted in the up-regulation of PI3K and p-AKT. One explanation could be that miR-132 diminished PETN, and indirectly activated the PI3K/AKT signaling pathway. Similar to the effect of overexpressing miR-132, PTEN inhibitor greatly enhanced cell proliferation, inhibited cell apoptosis, and repressed the expression of Bax and caspase-3/9 and inflammatory factors in chondrocytes. PTEN inhibitor antagonized the destructive effects from miR-132 inhibitor on PTEN/PI3K/AKT signaling pathway, which is in agreement with previous studies [28].

Wortmannin, the PI3K inhibitor [37], negatively affected the expression of PI3K, and inactivated the expression of p-AKT. It was reported that wortmannin inhibited cell growth and phosphorylation of AKT [38]. Therefore, PI3K inhibitor might pose a negative effect on cell proliferation through the inactivation of the AKT pathway. In the present study, suppression of PI3K significantly reduced cell proliferation, elevated cell apoptosis, and induced the expression of Bax and caspase-3/9, as well as inflammatory factors in chondrocytes after the down-regulation of miR-132. Inhibition of PI3K increased the destructive effect of miR-132 inhibitor on the PI3K/AKT signaling pathway, cell proliferation, and inflammation divisors of chondrocytes. These results could further support the role of PI3K/AKT in cell proliferation and apoptosis. However, there are still some limitations in our study. Due to the degenerative and inflammatory nature of OA, it is necessary to explore the catabolic index like MMP13, collagen II and inflammation cytokines such as IL-1β、IL-6、TNF-αin in further studies. On the other hand, it has been reported that downregulation of miR-132 inhibited the PI3K/Akt signaling and increased inflammation divisors in chondrocytes [34, 39]. Depending on the cell type, environment and stimulation manners, exogenous interventions might have complex interactions with physiological and pathological processes (apoptosis, inflammation, and etc.) by involving different molecules/signaling pathways (PI3K/AKT, NF-κB, and etc.). As a result, they can have different effects on cell survival and death. Therefore, it might be possible that a more complex network is involved in the regulation of OA by miR-132, which remains to be further explored.

## Conclusion

This study demonstrated that miR-132 was an important regulator of osteoarthritis in chondrocytes via regulation of the PTEN/PI3K/AKT signaling pathway.

## Data Availability

The datasets used and/or analyzed during the current study are available from the corresponding author on reasonable request.

## Abbreviations

OA: Osteoarthritis
miR-132: miRNA-132
miRNAs: MicroRNAs
PTEN: phosphatase and tensin homolog
